# Thyroiditis after mRNA Vaccination for COVID-19

**DOI:** 10.1155/2022/7604295

**Published:** 2022-11-09

**Authors:** Arunava Saha, Sanjita Chittimoju, Nitin Trivedi

**Affiliations:** ^1^PGY2 Internal Medicine, Saint Vincent Hospital, Worcester, MA, USA; ^2^Endocrinology and Metabolic Medicine, Saint Vincent Hospital, Worcester, MA, USA

## Abstract

**Background:**

SARS-CoV-2 has been known to cause multisystemic involvement, gaining entry through ACE-2 and TMPRSS2 receptors. COVID-19 vaccine-associated thyroiditis cases are now being reported. *Case Report*. *Case 1*. A 36-year-old woman with a history of right hemithyroidectomy for a benign thyroid nodule, on a stable dose of levothyroxine with euthyroid labs, presented with progressively worsening left neck pain, episodic palpitations, and heat intolerance after the second dose of mRNA1273 (Moderna) vaccine. Examination revealed an enlarged and tender left lobe of the thyroid with suppressed TSH but normal free T4 and ESR, signifying subacute thyroiditis. She was managed conservatively without corticosteroids or beta-blockers, and her symptoms resolved. A follow-up revealed increasing TSH, and levothyroxine was restarted. *Case 2*. A 33-year-old man with a history of anxiety disorder on Sertraline, presented with a two-week history of palpitations, heat intolerance, and 10-pound weight loss after the second dose of BNT162b2 (Pfizer-BioNTech) vaccine. Examination revealed a normal thyroid gland with no tenderness with elevated thyroid peroxidase and thyroglobulin antibodies. Ultrasound showed a diffusely heterogeneous thyroid with increased vascularity, suggesting silent thyroiditis. Follow-up revealed a hypothyroid phase with high TSH for which levothyroxine supplementation was started. *Discussion*. COVID-19 vaccine-associated subacute and silent thyroiditis have occurred following all three kinds of available vaccines, characterized by an initial thyrotoxic phase, followed by a hypothyroid phase and a recovery phase. Hypotheses include an immune response triggering thyroid inflammation or cross-reactivity with viral proteins.

**Conclusions:**

COVID-19 vaccine-associated thyroiditis is rare, but long-term monitoring of these patients is essential to ensure appropriate diagnosis and management of the potential hypothyroid phase.

## 1. Introduction

The novel coronavirus disease 2019 (COVID-19) caused by severe acute respiratory syndrome coronavirus-2 (SARS-CoV-2) has affected more than 250 million people worldwide [1]. It uses the angiotensin-converting enzyme 2 (ACE-2) and transmembrane protease serine-2 (TMPRSS2) receptors for entry that are expressed widely throughout the body including the endocrine glands. Hence, COVID-19 can have multisystemic involvement with a range of severity [2, 3].

Several effective vaccines have been introduced, and currently, mass vaccination drives are underway. The inactivated vaccines use adjuvants such as aluminum salts and toll-like receptor (TLR) agonists to increase the immunogenic response to vaccination [4]. However, in genetically susceptible individuals, adjuvants may lead to autoimmune/inflammatory syndrome induced by adjuvants (ASIA syndrome). Possible mechanisms include disrupting the immunological balance and molecular mimicry-triggering polyclonal activation of B lymphocytes [5]. Subacute thyroiditis following COVID-19 vaccination has been described with ASIA syndrome and also following mRNA and adenovirus vaccines that do not have an adjuvant.

We report two cases of thyroiditis that developed soon after receiving the second dose of the mRNA COVID-19 vaccine. The article's objective is to create awareness about a possible association between thyroiditis and the COVID-19 mRNA vaccines.

## 2. Case 1

A 36-year-old woman with a history of right hemithyroidectomy for a benign thyroid nodule six years ago, presented with a 6-week history of progressively worsening left-sided neck pain accompanied by palpitations and heat intolerance. She developed transient myalgia and neck pain after her first dose of mRNA1273 (Moderna COVID-19 vaccine). Her current symptoms started 2 weeks after receiving the second dose of the vaccine. She had been euthyroid on a stable dose of levothyroxine for the last 6 years. She denied any history of viral or respiratory illness prior to the onset of symptoms. She had no family history of thyroid disorders or autoimmune conditions. Laboratory evaluation revealed suppressed TSH at urgent care triggering a consultation with an endocrinologist, which took place in a few weeks, by when her symptoms were spontaneously resolving.

On physical examination, her heart rate was 88/min, the temperature was 37.7°C, her blood pressure was 110/65, and her respiratory rate was 20/min. A neck examination revealed an enlarged and tender left thyroid lobe. There was no palpable lymphadenopathy, exophthalmos, pharyngeal erythema, irregular heart rhythm, hepatosplenomegaly, edema, tremors, or skin lesions. Laboratory investigations revealed a TSH of less than 0.01 mIU/L with a free T4 of 1.8 ng/dl on the upper limit of normal (Table 1). Antithyroglobulin, anti-thyroid peroxidase, and antibodies to thyrotropin receptor were negative, and the erythrocyte sedimentation rate (ESR) was normal. She was diagnosed with subacute thyroiditis. Levothyroxine was held in view of suppressed TSH and elevated free T4. Corticosteroid treatment was not recommended given the mild nature of the symptoms. Her symptoms gradually subsided over the subsequent few weeks, and TSH levels started rising. Levothyroxine therapy was restarted when the TSH level increased above the normal range.

## 3. Case 2

A 33-year-old man with a past history of anxiety disorder treated with sertraline presented with a two-week history of persistent palpitations, heat intolerance, and 10 pounds of weight loss. He reported no past or family history of thyroid disorders. He did not have any history of COVID-19 infection and was vaccinated with both doses of BNT162b2 (Pfizer-BioNTech COVID-19 vaccine) 4 weeks apart. Soon after receiving the second vaccine dose, he developed a high-grade fever at 102.5 F, myalgia, and sore throat, which persisted for 2 weeks. Subsequently, he experienced palpitations, diaphoresis, and heat intolerance along with generalized weakness. An endocrinology referral was triggered as he was found to have suppressed TSH along with elevated CRP at his primary care provider's office (Table 1). On physical examination, his heart rate was 86/min, blood pressure was 143/71, and respiratory rate was 18. A thyroid examination revealed a normal thyroid gland with no significant tenderness. He had no extrathyroidal features of Graves' disease. Repeat laboratory investigations revealed an undetectable TSH with normal T3 and T4 levels. Additional lab investigations revealed elevated thyroid peroxidase and thyroglobulin antibodies but undetectable levels of thyroid receptor antibodies and thyroid-stimulating immunoglobulin antibodies. An ultrasound of the thyroid showed a diffusely heterogeneous gland with increased vascularity and no nodules. The clinical features were consistent with silent thyroiditis. There were a few large benign-appears lymph nodes, which were resolved on follow-up ultrasound. His symptoms improved with NSAIDs without corticosteroid treatment. Repeat laboratory evaluation 4 weeks later revealed elevated TSH and low free T4 suggestive of a hypothyroid phase. He was transiently treated with levothyroxine because of severe fatigue and relative bradycardia with a heart rate of 50 s. Levothyroxine was subsequently discontinued as his TSH normalized. He currently remains euthyroid and asymptomatic without levothyroxine supplementation.

## 4. Discussion

We describe two cases of thyroiditis associated with the COVID-19 vaccine, subacute thyroiditis for the first case and silent thyroiditis for the second case. Both patients had an initial thyrotoxic phase followed by a hypothyroid phase which required levothyroxine supplementation.

Subacute and silent thyroiditis is a self-limiting thyroid disorder. They are more prevalent in women. Viral infections and immune-modulatory drugs have been implicated as etiological factors. Characteristically, they have an initial destructive thyrotoxic phase, followed by a hypothyroid phase and a recovery phase, leading to the normalization of thyroid functions [6].

The endocrine system has been found to be involved in COVID-19, causing hypopituitarism, adrenal necrosis, pancreatic beta-cell damage, gonadal dysfunction, and thyroiditis [7]. The exact pathogenesis is unknown, but the hypothesis involves an immune-mediated hormonal dysfunction, direct virus entry into cells causing cell destruction or cellular dysfunction caused by inflammation [7, 8].

Along with the common viruses such as CMV and HSV, cases of thyroiditis have emerged in association with COVID-19. The ACE-2 and TMPRSS2 that allow SARS-CoV-2 to infiltrate human cells are highly expressed in the thyroid follicular cells [9].

A recent study by Muller et al. suggested silent thyroiditis induced by COVID-19 occurs in an underlying setting of nonthyroidal illness in critically ill male patients. This was usually followed by a permanent hypothyroid phase [10]. However recent systematic reviews have demonstrated several cases of subacute thyroiditis secondary to COVID-19 infection, amongst which 92% presented with neck pain and the majority were female patients [11, 12]. This suggests that thyroiditis caused by COVID-19 infection can have varied clinical presentations.

Additionally, an increasing number of cases of COVID-19 vaccine-associated thyroiditis are now being reported. These cases have occurred following all three kinds of available vaccines, inactivated SARS-CoV-2 vaccine, mRNA-based vaccine, and vector-based vaccine. Most cases that developed after the inactivated vaccine have been attributed to adjuvants, triggering adverse reactions in genetically predisposed individuals, and causing ASIA syndrome [13–15]. In a case report of two cases of subacute thyroiditis postvaccination from Germany, symptoms were attributed to the adenovirus vector vaccine and the mRNA vaccine. Both the patients had classic symptoms and fine-needle aspiration cytology showed lymphocytic infiltrate and multinucleated giant cells consistent with subacute thyroiditis [16].

A few other cases have been described, including a 42-year-old female who developed painful hyperthyroid symptoms five days after the first dose of the BNT162b2 vaccine and required a short course of steroids and beta-blockers with which her symptoms improved [15]. Similarly, a 46-year-old female developed neck pain after the second dose of the Moderna vaccine and was diagnosed to have subacute thyroiditis. She was managed with analgesics, oral steroids, and beta-blockers and was found to have normal thyroid function at follow-up [17].

Adenovirus-vector vaccines stimulate innate immunity sensors whereas mRNA vaccines encode the viral spike S glycoprotein, causing a potent immunological response. The vaccine is formulated in lipid particles which enable the delivery of RNA into the host cells to allow expression of the SARS-CoV-2 S antigen. This process elicits an immune response to the S antigen, which protects against COVID-19 infection [18].

The mechanism of postvaccination-associated thyroiditis remains unknown. It has been hypothesized that vaccinations may be responsible for producing an immune response that triggers thyroid inflammation in predisposed patients. Another hypothesis includes the SARS-CoV-2 spike protein, nucleoprotein, and membrane protein which cross-reacts with thyroid peroxidase [19].

There has been some recent understanding regarding the potential immune upheaval due to mRNA COVID-19 vaccination and its role in causing myocarditis, particularly in young men. The mechanism may include molecular mimicry between the spike protein of SARS-CoV-2 and self-antigens, increased cytokine expression secondary to dysregulated immune pathways, or an exaggerated immune response to viral mRNA [20].

In these two cases of thyroiditis after the mRNA COVID-19 vaccination, although a direct causal relationship could not be established, however, no other causes could be identified and temporality with the administration of the mRNA COVID-19 vaccine is very strongly suggestive. Up to 10% of patients with subacute thyroiditis develop permanent hypothyroidism. Hence, it is important that these patients are followed long-term [21]. Overall, further data are needed to determine the association between subacute thyroiditis and COVID-19 vaccinations.

## 5. Conclusion

COVID-19 vaccine-induced thyroiditis can range from subacute to silent presentation and is a rare phenomenon. The purpose of this report is to raise awareness among providers about the same. It is important for clinicians to be aware of this presentation to recognize COVID-19 vaccine-related immune effects in the thyroid to accurately manage the relevant stages of thyroiditis. Long-term monitoring of these patients is essential to ensure an appropriate diagnosis of the potential hypothyroid phase in them. Further investigation is needed to determine possible predisposing factors and mechanisms leading to thyroiditis after the COVID-19 vaccine.

## Figures and Tables

**Table 1:** Laboratory test results.

	Case 1	Case 2
At diagnosis		
TSH (0.35–4.94 mIU/L)	<0.01	<0.01
FT4 (0.9–1.8 ng/dl)	1.8	1.71
T3 (100–200 ng/dl)	188	139
Anti-TPO (<10 IU/ml)	7	147
Anti-TG (<14 IU/ml)	Negative	1296.1
TRAB (<1.75 IU/L)	<1.10	<1.10
ESR (<20 mm/hr)	2	—
TSI antibodies	—	<0.10
CRP (<5.0 mg/L)	—	25
WBC (4.0–11.0) ×10^3^/ml	—	5.3
Follow-up 4^th^ week		
TSH	14.23	25
FT4	0.90	0.40
T3	—	—

## Data Availability

The data used to support the findings of this study are included within the article.
